# Association between recognition and help-seeking preferences and stigma towards people with mental illness

**DOI:** 10.1017/S2045796016000998

**Published:** 2016-12-08

**Authors:** L. Picco, E. Abdin, S. Pang, J. A. Vaingankar, A. Jeyagurunathan, S. A. Chong, M. Subramaniam

**Affiliations:** Research Division, Institute of Mental Health, Singapore

**Keywords:** Help-seeking, mental health literacy, recognition, Singapore, stigmatising attitudes

## Abstract

**Aims.:**

The ability to recognise a mental illness has important implications as it can aid in timely and appropriate help-seeking, and ultimately improve outcomes for people with mental illness. This study aims to explore the association between recognition and help-seeking preferences and stigmatising attitudes, for alcohol abuse, dementia, depression, obsessive-compulsive disorder (OCD) and schizophrenia, using a vignette-based approach.

**Methods.:**

This was a population-based, cross-sectional survey conducted among Singapore Residents (*n* = 3006) aged 18–65 years. All respondents were asked what they think is wrong with the person in the vignette and who they should seek help from. Respondents were also administered the Personal and Perceived sub scales of the Depression Stigma Scale and the Social Distance Scale. Weighted frequencies and percentages were calculated for categorical variables. A series of multiple logistic and linear regression models were performed separately by vignette to generate odd ratios and 95% confidence intervals for the relationship between help-seeking preference, and recognition and beta coefficients and 95% confidence intervals for the relationship between stigma and recognition.

**Results.:**

Correct recognition was associated with less preference to seek help from family and friends for depression and schizophrenia. Recognition was also associated with increased odds of endorsing seeking help from a psychiatric hospital for dementia, depression and schizophrenia, while there was also an increased preference to seek help from a psychologist and psychiatrist for depression. Recognition was associated with less personal and perceived stigma for OCD and less personal stigma for schizophrenia, however, increased odds of social distancing for dementia.

**Conclusion.:**

The ability to correctly recognise a mental illness was associated with less preference to seek help from informal sources, whilst increased preference to seek help from mental health professionals and services and less personal and perceived stigma. These findings re-emphasise the need to improve mental health literacy and reinforce the potential benefits recognition can have to individuals and the wider community in Singapore.

## Introduction

Mental health literacy refers to ‘knowledge and beliefs about mental disorders, which aid their recognition, management or prevention’ (Jorm *et al.* 1997). It comprises several components: (i) the ability to recognise specific disorders; (ii) knowledge and beliefs about risk factors and causes of mental disorders as well as awareness of professional help available; (iii) attitudes which facilitate recognition and appropriate help-seeking; and (iv) how to access mental health information (Jorm, 2000). The inability to correctly recognise mental disorders can result in inappropriate help-seeking and delays in treatment seeking (Jorm, 2000). Furthermore, studies have shown that delays in seeking appropriate treatment result in negative outcomes, due to longer duration of untreated illness, and consequently, poorer treatment outcomes (Marshall *et al.* 2005; Altamura *et al.* 2008, 2010; de Diego-Adeliño *et al.* 2010).

Whilst there are several factors that may determine or influence help-seeking, recognition plays a key role in this process (Biddle *et al.* 2007; Gulliver *et al.* 2010). Despite this, there has been much debate about the positive and negative implications of using psychiatric terms by the public to label someone with a mental disorder (Angermeyer & Matschinger, 2005). With regards to the potentially harmful effects, it has been argued that labelling creates expectations of deviant behaviour (Wright *et al.* 2007) and may increase stigma (Jorm & Griffiths, 2008). However, being able to identify or recognise the signs and symptoms of mental illness is linked to early help-seeking and can reduce the burden of disease associated with mental disorders (Wang *et al.* 2005).

Several studies amongst young people and adult populations have explored the relationship between recognition or labelling and help-seeking preferences and intentions. Among Australian adolescents, research findings suggest that recognition of mental disorders in vignettes predicted a preference for recommending or intending to seek help from professional sources, whilst also being associated with less preference to seek help from informal sources such as family and friends (Wright *et al.* 2012; Yap *et al.* 2014). Similar findings have been found in adult populations (Angermeyer *et al.* 2009; Rusch *et al.* 2012); however, the majority of these studies have focused on depression, schizophrenia and related psychoses and social phobia. Hence less is known about other mental illnesses such as obsessive-compulsive disorder (OCD) or alcohol abuse.

Whilst recognition can influence and affect when and where help is sought, it is also related to stigma. The detrimental effects of stigma are widespread as it can act as a barrier to help-seeking and service use as well as achievement of age-appropriate functional goals (Corrigan *et al.* 2009; Rüsch *et al.* 2009; Clement *et al.* 2015). There seems to be little consensus however, regarding the relationship between recognition and stigma. For example, mental health literacy interventions have been found to reduce stigma, with some studies finding those with greater knowledge of mental illness, were less likely to hold stigmatising attitudes (Brockington *et al.* 1993; Thornton & Wahl, 1996; Holmes *et al.* 1999; Kitchener & Jorm, 2006). Others however, have determined that having knowledge to be able to label someone as mentally ill is in fact associated with increased stigma (Martin *et al.* 2000; Angermeyer & Matschinger, 2004; Peluso & Blay, 2009; Hengartner *et al.* 2013).

Link and Phelan (2010), however, argue that recognition or labelling has both positive and negative aspects and that whilst labelling a person as ‘mentally ill’ can be stigmatising, labelling the problem or the illness itself, can be beneficial, as it facilitates treatment and ultimately amelioration of symptoms. Given the ambiguity and lack of clarity surrounding recognition and stigma, there is a need to further examine mental health literacy and more specifically recognition and stigma together, as the negative stereotypes implicit in stigma involve distortions of knowledge and understanding (Holman, 2015).

Very little is known about the relationships between recognition, help-seeking and stigma in non-Western settings, and more specifically in Singapore, a multi-ethnic city–state situated in South East Asia. Singapore has an urban population of approximately 5.5 million, comprising predominantly of three main ethnic groups: Chinese, Malays and Indians (Statistics Singapore, 2015). The recently completed Mind Matters Study was the first national mental health literacy study of its kind in Singapore, which found significant differences in terms of recognition across five mental disorders. The most well-recognised disorders were dementia (66.3%), alcohol abuse (57.1%) and depression (55.2%), while only 28.7 and 11.5% could recognise OCD and schizophrenia, respectively (Chong *et al.* 2016). Previous mental health literacy interventions often follow the premise that recognition of a problem is the first step prior to appropriate help-seeking (Wright *et al.* 2007). Given that recognition can aid in timely and appropriate help-seeking, and ultimately improve outcomes for people with mental illness, the relationship between recognition and other outcomes warrants further investigation. The aim of the current study was to explore the association between recognition and how this may predict help-seeking preferences and stigmatising attitudes, for alcohol abuse, dementia, depression, OCD and schizophrenia, using a vignette-based approach, among Singapore Residents aged 18–65 years. Based on existing literature, we hypothesised that being able to recognise mental disorders would be associated with: (i) greater preference to seek help from mental health professionals; (ii) less preference to seek help from informal sources such as family and friends; and (iii) would be associated with less personal stigma towards people with mental illness.

## Methods

### Participants and procedure

The Mind Matters Study was a comprehensive, population-based, cross-sectional mental health literacy survey conducted among Singapore Residents (Citizens and Permanent Residents) aged 18–65 years, who were living in Singapore between March 2014 and April 2015 (Chong *et al.* 2016). The sample was derived using the sampling frame from an administrative database in Singapore that maintains data on age, gender, ethnicity and residential address of all those residing in Singapore. Residents living outside of Singapore, those who were unable to be contacted due to incomplete or incorrect addresses and those who were unable to complete the interview in one of the specified languages were excluded from the survey. Trained interviewers administered the survey in English, Mandarin, Malay or Tamil, based on the respondent's preference and on average the interview took 45 min to complete. The study was approved by the institutional ethics review board (National Healthcare Group Domain Specific Review Board). All respondents provided written informed consent and in the case of a respondent who was aged below 21 years, the written informed consent was also obtained from a legally acceptable representative, parent or guardian. A total of 3006 people completed the face-to-face interview, resulting in an overall response rate of 71%.

### Survey interview

The interview was based on a vignette describing a person with one of five mental disorders; alcohol abuse, dementia, depression, OCD or schizophrenia, and followed a similar protocol to those used in previous research (Jorm *et al.* 1997). Respondents were randomly allocated one of the five vignettes and were matched by gender and ethnicity, whereby they were read a vignette about someone of the same gender and ethnicity as them. All vignettes were written to reflect the diagnostic criteria for the five disorders according to Diagnostic and Statistical Manual of Mental Disorders, 4th Edition (DSM-IV) and International Statistical Classification of Diseases and Related Health Problems, 10th Edition (ICD-10). The vignettes and related questions, along with the other measures included in the questionnaire, were pre-tested to ensure an adequate level of understanding among the local population and adaptations were made where needed, prior to the commencement of the survey.

The interview began by collecting socio-demographic information relating to the respondents including their age, gender, ethnicity, marital status, highest education-level attained, employment status and monthly income, using a structured questionnaire. Following this they were read one of the five vignettes and were asked the following open text questions: ‘What do you think the person in the vignette is suffering from?’ and ‘Who do you think the person in the vignette should seek help from?’ Respondents were also asked if they feel these symptoms were a cause for concern (yes/no) and whether they or someone close to them had experienced problems similar to those in the vignette.

Respondents were administered two stigma measures. The first was the Depression Stigma Scale which consists of two subscales, one which measures personal stigma and the other, perceived stigma (Griffiths *et al.* 2004). The personal stigma subscale measures stigma in the respondent's own attitudes towards the person in the vignette, while the perceived stigma subscale assesses the respondent's beliefs about the attitudes of others towards the person in the vignette. Both subscales consist of nine items, however one item (‘I would not vote for a politician if I knew they had a mental illness’) was excluded. Responses to each item are measured on a five-point scale, ranging from ‘strongly disagree’ to ‘strongly agree’. Whilst the scale was originally intended to measure stigma related to depression, it can also be administered in relation to other disorders (Griffiths *et al.* 2006). Scores for each subscale were calculated by summing the respective item scores, with higher scores indicating greater personal or perceived stigma. The second stigma measure was the Social Distance Scale which measures self- reported willingness to have contact with, or the desire to socially distance oneself from the person described in the vignette (Link *et al.* 1999). The scale consisted of five items ranked on a four-point scale from definitely willing to definitely unwilling, where scores were calculated by summing item scores and higher scores indicated greater social distance.

### Coding and content analysis of open-ended questions

Coding of the open text questions relating to recognition and recommended help-seeking sources were conducted by senior members of the study team. Regarding recognition, responses were coded as correct if the respondent was able to correctly label or name the specific condition. Where the response was a near approximation of the correct answer, two senior investigators (M.S. and S.A.C.) would come to a consensus on how that response should be coded (Chong *et al.* 2016). Sources of help-seeking were broadly coded as follows: ‘talk to family or friends,’ ‘seek help from the Institute of Mental Health (IMH)’ (the only tertiary psychiatric hospital dedicated to providing mental health services in Singapore), ‘see a psychologist,’ ‘see a psychiatrist,’ ‘see a doctor or general practitioner (GP),’ and ‘see a counsellor or have counselling,’ (Picco *et al.* 2016). Those responses endorsed by <3% of respondents were categorised as ‘other’, however given the heterogeneity of these responses, they were not included in this analysis.

### Statistical analysis

All estimates were weighted to adjust for over sampling and post-stratified for age and ethnicity distributions between the survey sample and the Singapore resident population in the year 2012. Weighted frequencies and percentages were calculated for categorical variables. A series of multiple logistic and linear regression models were performed separately by vignette to generate odd ratios (ORs) and 95% confidence intervals for the relationship between help-seeking preference (dependent variable) and recognition (main predictor variable) and beta coefficients and 95% confidence intervals for the relationship between stigma (dependent variable) and recognition (main predictor variable), after adjusting for age, gender, ethnicity, marital status, education, employment, income as well as exposure to mental health problems either in themselves and/or family or friends. The six most frequently reported help-seeking sources, which comprised seeking help from family and friends, IMH, psychologist, doctor or GP, counsellor and psychiatrist (Picco *et al.* 2016), as well as three dimensions of stigma (personal stigma, perceived stigma and social distance) were included as dependent variables. In these regression analyses, all six help-seeking preferences were treated as binary dependent variables and the three dimensions of stigma were treated as continuous dependent variables. Standard errors (s.e.) and significance tests for survey data analysis procedures were estimated using the Taylor series’ linearisation method to adjust for the weighting. Multivariate significance was evaluated using Wald *χ*^2^ tests based on design corrected coefficient variance–covariance matrices. Statistical significance was evaluated at the 0.05 level using two-sided tests. Data analysis was conducted using the SAS Version 9.3.

## Results

The socio-demographic characteristics of the respondents are shown in Table 1. The sample comprised almost equal proportions of females (49.9%) and males (50.1%). The majority of the sample was of Chinese ethnicity (74.7%) and married (64%). 95% of people felt the symptoms described in the vignette were a cause for concern or worry, while 43.7% could identify the condition being described in the vignette.

**Table 1.** *Descriptive statistics of the sample (*n = *3006)*

	*N*	Weighted %	s.e.
Age group			
18–34 years	1152	34.4	0.04
35–49 years	896	35.2	0.04
50–65 years	958	30.5	0.1
Gender			
Female	1506	49.1	1.3
Male	1500	50.9	1.3
Ethnicity			
Chinese	1034	74.7	0.04
Malay	977	12.8	0.01
Indian	963	9.1	0.01
Others	32	3.3	0.04
Marital status			
Married	1916	64.0	1.0
Never married	927	31.4	0.9
Others (divorced, widowed, separated)	162	4.6	0.5
Education			
Primary education and below	431	13.4	0.8
Secondary education include O/N level*	820	25.8	1.0
A level, polytechnic and other diploma	999	31.3	1.1
University	756	29.6	1.1
Employment status			
Employed	2227	77.6	1.0
Housewife/homemaker	378	8.7	0.6
Retired	78	3.0	0.4
Student	203	6.7	0.5
Unemployed	120	3.9	0.5
Income			
<SGD2000	1346	40.5	1.2
SGD2000–5999	1162	46.4	1.3
SGD6000 and above	294	13.1	0.9
Vignette type			
Alcohol Abuse	603	20.8	1.0
Dementia	596	19.2	1.0
Depression	607	19.9	1.0
Obsessive-compulsive disorder (OCD)	605	20.5	1.0
Schizophrenia	595	19.6	1,0
Ever had problems similar to subject	319	11.4	0.8
Family or friends ever had problems similar to subject	700	22.6	1.1
Symptoms a cause for concern or worry	2860	95.0	0.5
Recognition (overall sample)	1173	43.7	1.2
Alcohol abuse	339	57.1	2.7
Dementia	300	66.3	2.6
Depression	317	55.2	2.8
OCD	157	28.7	2.5
Schizophrenia	60	11.5	1.8

* Equivalent to up to 11 years of education.

Table 2 shows the association between recognition and help-seeking preferences by each vignette. It shows that recognition was associated with less preference to seek help from family and friends for depression and schizophrenia. Recognition was also associated with increased odds of endorsing seeking help from IMH (a tertiary psychiatric hospital in Singapore) for dementia, depression and schizophrenia while there was also an increased preference to seek help from a psychologist and psychiatrist for depression.

**Table 2.** Association between recognition and help-seeking preference by vignette

	Alcohol abuse			Dementia			Depression			OCD			Schizophrenia		
	OR*	95% CI		OR*	95% CI		OR*	95% CI		OR*	95% CI		OR*	95% CI	
Family and friends	0.62	0.37	1.06	0.57	0.30	1.11	**0**.**42**	**0**.**25**	**0**.**70**	0.39	0.15	1.04	**0**.**25**	**0**.**08**	**0**.**84**
IMH				**5.56**	**1.97**	**15.71**	**8.50**	**2.09**	**34.51**	1.30	0.41	4.14	**5.66**	**2.07**	**15.51**
Psychologist	0.65	0.20	2.11	0.10	0.01	1.72	**10.32**	**1.70**	**62.77**	0.88	0.36	2.18	1.15	0.23	5.91
Doctor/GP	1.72	0.79	3.77	1.24	0.72	2.13	1.46	0.79	2.69	0.57	0.27	1.21	0.76	0.30	1.91
Counsellor	0.81	0.37	1.74	1.21	0.44	3.35	1.21	0.51	2.83	3.68	0.91	14.82	0.43	0.05	3.34
Psychiatrist	1.13	0.48	2.65	0.84	0.40	1.77	**3.51**	**1.39**	**8.83**	1.26	0.63	2.50	2.22	0.96	5.11

Reference = incorrect recognition.

* =Odds ratio (OR) was derived using multiple logistic regression analyses after adjusting for age, gender, ethnicity, marital status, education, employment, income as well as exposure to mental health problems either in themselves and/or family or friends.

Bold figures indicate a significant result at the *p* < 0.05 level.

Table 3 shows the association between recognition and the three types of stigma; personal stigma, perceived stigma and social distance. Findings revealed that recognition was associated with less personal and perceived stigma for OCD and less personal stigma for schizophrenia. Recognition was also associated with increased odds of social distancing for dementia.

**Table 3.** Association between recognition and stigma by vignette

	Alcohol abuse			Dementia			Depression			OCD			Schizophrenia		
Stigma	Beta*	95% CI		Beta*	95% CI		Beta*	95% CI		Beta*	95% CI		Beta*	95% CI	
Personal stigma	−0.05	−0.85	0.75	−0.45	−1.36	0.46	−0.33	−1.12	0.46	**−2**.**12**	**−3**.**01**	**−1**.**24**	**−2**.**25**	**−3**.**21**	**−1**.**29**
Perceived stigma	−0.06	−0.69	0.58	−0.01	−0.59	0.57	0.45	−0.16	1.06	**−1.05**	**−1.72**	**−0.37**	−0.05	−1.25	1.14
Social distance	0.56	−0.23	1.35	**1.43**	**0.45**	**2.42**	−0.04	−0.93	0.86	0.03	−1.03	1.08	−0.39	−1.51	0.72

Reference = incorrect recognition.

* =Beta coefficient (Beta) was derived using multiple linear regression analyses after adjusting for age, gender, ethnicity, marital status, education, employment, income as well as exposure to mental health problems either in themselves and/or family or friends.

Bold figures indicate a significant result at the *p* < 0.05 level.

## Discussion

The findings from this study have highlighted some important associations between the ability to correctly recognise a mental disorder and help-seeking preferences and stigmatising attitudes. For depression and schizophrenia, recognition was associated with less preference to seek help from family and friends while higher odds of seeking help from professional sources such as a psychiatrist, psychologist or a psychiatric hospital, for depression. While recognition was associated with less personal and perceived stigma for OCD and less personal stigma for schizophrenia, it was related to greater social distancing for dementia.

As hypothesised, the odds of seeking help from informal sources such as friends and family were lower for those who could correctly recognise depression and schizophrenia. In a study among Australians aged 12–25 years, recognition was associated with less preference to seek help from friends (Wright *et al.* 2012). Given that mental health literacy encompasses the ability to be able to recognise and identify mental disorders as well as having an awareness of professional help available (Jorm, 2000) it is not surprising recognition was associated with less preference to seek help from family and friends. Interestingly, among the same sample, the most commonly reported source of help for depression and schizophrenia was family and friends (Picco *et al.* 2016) and therefore this suggests that the ability to recognise or label a disorder is influential in terms of help-seeking preferences. It is also important to note that the upper limit of the confidence interval for alcohol abuse, dementia and OCD was 1.06, 1.11 and 1.04, respectively (while the lower limits were all greater than zero), which does suggest that, although these findings are not significant, they are borderline and therefore it appears there is a similar trend across all five disorders, where correct recognition is associated with less preference to seek help from informal sources.

Recognition was associated with increased odds in seeking help from IMH for depression, dementia and schizophrenia. IMH is a 2000 bed acute tertiary psychiatric hospital, with outpatient clinics, which offers a comprehensive range of psychiatric, rehabilitative and counselling services for children, adolescents, adults and the elderly. Since its inception nearly 90 years ago, IMH has grown to not only provide clinical services, but also community-based services and support services for patients and their caregivers. It is thus well known both as an Institution that provides care for those with psychiatric illness and for the availability of trained mental health professionals and a wide range of services in Singapore. Therefore it is logical that those who recognised the conditions correctly were able to recommend seeking services from this Institution.

For depression, the preference to seek help from mental health professionals such as psychologists or psychiatrists was strongly associated with recognition. This finding is based on open text responses where respondents indicated who or where they would recommend the person in the vignette should seek help from. In order to articulate and name specific professionals, this requires a level of knowledge and understanding and therefore it is not surprising to find those who can recognise depression are also more likely to suggest help-seeking from mental health professionals. Beliefs about mental illness aetiology may also be an additional influencing factor relating to help-seeking preferences, whereby despite correctly recognising the disorder based on the vignette, respondents had differing beliefs about the causes of these mental illnesses and accordingly this also influenced their help-seeking preferences (Rusch *et al.* 2012; Schomerus *et al.* 2012). Aetiology beliefs could also be culturally influenced (Chen & Mak, 2008) and therefore this gives merit to further investigate the impact of such beliefs on help-seeking preferences.

Recognition appears to be one of the influencing factors relating to help-seeking preferences; correct recognition was associated with less odds in recommending help-seeking from informal sources, whilst higher odds in recommending help-seeking from mental health professionals and services such as psychiatrists, psychologists and IMH. These findings are important as they suggest that those who can correctly identify the signs and symptoms for specific mental disorders are more likely to recommend help-seeking from mental health professionals. Despite this, it is also important to acknowledge that the relationship between mental health literacy and help-seeking is complex and multi-factorial and therefore, help-seeking preferences or behaviours may not solely be influenced by mental health literacy or the ability to recognise signs and symptoms, but additional factors such as aetiology beliefs, cultural influences, illness severity or stigma.

Mental health literacy studies in various countries have shown that failure to recognise mental disorders is not uncommon (Jorm *et al.* 1997; Wong & He, 2011; Chong *et al.* 2016) although, recognition has improved over time, particularly for schizophrenia (Jorm *et al.* 2006*b*; Angermeyer *et al.* 2009; Reavley & Jorm, 2011). Despite this, social rejection and stigmatising attitudes towards people with mental illness has remained fairly stagnant over the past 20 years. The systematic review and meta-analysis by Schomerus *et al.* (2012) found that whilst mental health literacy has improved, negative attitudes towards people with a mental illness have not and even more concerning was that attitudes towards schizophrenia have in fact worsened over time.

Contrary to this, we found those who were able to identify OCD and schizophrenia displayed less personal stigma, as well as less perceived stigma towards OCD. It is possible that recognition or greater mental health literacy in terms of awareness and understanding, would therefore result in people not only being more aware of the available treatment options, but also the improved outcomes that are associated with treatment seeking, causing people to hold less stigmatising views. A previous study among the same population found that recognition was associated with higher levels of education (Chong *et al.* 2016), while research has shown that people with increased knowledge about mental illness are less likely to endorse stigmatising attitudes (Holmes *et al.* 1999; Subramaniam *et al.* 2016) and this may further explain this finding.

With regards to social distance, contrasting results were found; recognition was associated with greater social distancing towards dementia. Social distance was assessed by asking the respondent how willing they would be to spend an evening, make friends, and work closely with the person in the vignette as well as have this person move next door and have them marry into their family. Given the person in the dementia vignette was aged 75, it is not surprising that the majority of people were unwilling to work with this person and have them marry into their family. Upon further analysis we actually found that if these items were removed, social distancing was no longer significantly associated with correct recognition and this may therefore explain why we observed greater social distancing towards dementia. To date, the majority of research surrounding recognition and social distancing has related to schizophrenia or depression, and results have been mixed (Jorm & Griffiths, 2008; Angermeyer *et al.* 2009). Further research exploring the impacts of recognition and stigma on other mental disorders is therefore needed.

Our findings should be viewed in light of the following limitations. The cross sectional nature of the study precludes any causal inferences and therefore associations could be bidirectional. Respondents were asked to identify what the person in the vignette was suffering from and recommend where they should seek help from; however, recognition of symptoms and help-seeking intentions may not necessarily equate to help-seeking behaviour (Jorm *et al.* 2000; Barney *et al.* 2006). Thus questionnaires that examine intended behaviours should be considered in future surveys. It is possible that respondents may have provided socially desirable responses rather than their preferred responses. Finally, whilst a representative sample was used, non-responders may have differing views, beliefs and knowledge relating to mental disorders.

In summary, findings from the study suggest that recognition was associated with less preference to seek help from informal sources such as family and friends and increased preference to seek help from mental health professionals and services including psychiatrists, psychologists and IMH. Given that less than half of the general population could recognise mental disorders based on vignettes (Chong *et al.* 2016), this re-emphasises the need to improve mental health literacy amongst the local population in Singapore. Furthermore, given that recognition was associated with less personal and perceived stigma, this reinforces the potential benefits recognition can have to individuals and the wider community. It has been shown that even small increases in knowledge can have a significant effect on overall attitudes and behaviour (Wolff *et al.* 1996), and therefore educating the public about mental illness, the signs and symptoms and the importance of appropriate and timely help-seeking is crucial.

Moving forward, recognition of mental disorders needs to be a focus of mental health community awareness initiatives designed to facilitate help-seeking (Dumesnil & Verger, 2009), especially given that recognition is generally poorer when compared with Western countries (Jorm *et al.* 2006*a*; Angermeyer *et al.* 2009; O'Keeffe *et al.* 2016). Going beyond this, as knowledge is not the only influencing factor relating to help-seeking, interventions could also address issues relating to stigma and implications of delaying help-seeking (Klineberg *et al.* 2011). Mental health literacy interventions have been shown to have an impact on reducing social distance and stigma (Christensen *et al.* 2004; Kitchener & Jorm, 2006). Given the extent of stigma towards those with mental illness (Subramaniam *et al.* 2016), and the lack of such interventions in the local context, this further emphasises the need for initiatives to address mental health literacy and stigma at the population level, in the future.

Given the complex inter-related relationship between recognition, beliefs, help-seeking preferences and stigmatising attitudes, there is a need for more in-depth qualitative studies to delve further into the subjective and inter-personal processes of these intertwined constructs. Furthermore, whilst changing or improving knowledge and beliefs about mental illness can influence behaviour, little is known about whether behaviour changes are actually made and moving forward, there is a need to track and monitor help-seeking behaviour over time.

## Availability of Data and Materials

Data are not available for online access; however, readers who wish to gain access to the data can write to the senior author Dr Mythily Subramaniam at mythily@imh.com.sg with their requests. Access can be granted subject to the Institutional Review Board (IRB) and the research collaborative agreement guidelines. This is a requirement mandated for this research study by our IRB and funders.
